# Validation of Visually Identified Muscle Potentials during Human Sleep Using High Frequency/Low Frequency Spectral Power Ratios

**DOI:** 10.3390/s22010055

**Published:** 2021-12-22

**Authors:** Mo H. Modarres, Jonathan E. Elliott, Kristianna B. Weymann, Dennis Pleshakov, Donald L. Bliwise, Miranda M. Lim

**Affiliations:** 1 Mental Illness Research, Education and Clinical Center (MIRECC-VISN1), VA Bedford Health Care System, Bedford, MA 01730, USA; mo.modarres@va.gov; 2 VA Portland Health Care System, Portland, OR 97239, USA; elliojon@ohsu.edu; 3 Department of Neurology, Oregon Health & Science University, Portland, OR 97239, USA; 4 School of Nursing, Oregon Health & Science University, Portland, OR 97239, USA; weymannk@ohsu.edu; 5 School of Medicine, Oregon Health & Science University, Portland, OR 97239, USA; pleshako@ohsu.edu; 6 Department of Neurology, Emory University, Atlanta, GA 30322, USA; dbliwis@emory.edu; 7 National Center for Rehabilitative Auditory Research, VA Portland Health Care System, Portland, OR 97239, USA; 8 Department of Behavioral Neuroscience, Oregon Health & Science University, Portland, OR 97239, USA; 9 Oregon Institute of Occupational Health Sciences, Oregon Health & Science University, Portland, OR 97239, USA

**Keywords:** electromyography, EMG, polysomnography, REM sleep without atonia, REM sleep behavior disorder, RBD, parkinsonism, Parkinson’s disease, spectral power

## Abstract

Surface electromyography (EMG), typically recorded from muscle groups such as the mentalis (chin/mentum) and anterior tibialis (lower leg/crus), is often performed in human subjects undergoing overnight polysomnography. Such signals have great importance, not only in aiding in the definitions of normal sleep stages, but also in defining certain disease states with abnormal EMG activity during rapid eye movement (REM) sleep, e.g., REM sleep behavior disorder and parkinsonism. Gold standard approaches to evaluation of such EMG signals in the clinical realm are typically qualitative, and therefore burdensome and subject to individual interpretation. We originally developed a digitized, signal processing method using the ratio of high frequency to low frequency spectral power and validated this method against expert human scorer interpretation of transient muscle activation of the EMG signal. Herein, we further refine and validate our initial approach, applying this to EMG activity across 1,618,842 s of polysomnography recorded REM sleep acquired from 461 human participants. These data demonstrate a significant association between visual interpretation and the spectrally processed signals, indicating a highly accurate approach to detecting and quantifying abnormally high levels of EMG activity during REM sleep. Accordingly, our automated approach to EMG quantification during human sleep recording is practical, feasible, and may provide a much-needed clinical tool for the screening of REM sleep behavior disorder and parkinsonism.

## 1. Introduction

The unambiguous determination of rapid eye movement (REM) sleep relies on the simultaneous collection of electroencephalography, electrooculography, and electromyography (EMG; conventionally via mentalis/submentalis activity) [1]. One hallmark neurophysiologic feature of REM sleep is skeletal muscle paralysis (outside of specific ventilatory musculature, e.g., the diaphragm) reflected by relatively low EMG voltage at or near detectable noise levels [2,3]. Early methods of EMG quantification relied on analog-to-digital hardware and were limited to comparatively basic functions composed of summating signal voltage in an effort to objectively discriminate between REM and NREM [4].

Renewed efforts in the field of EMG signal analysis and quantification came after the first description of REM sleep behavior disorder (RBD), characterized by abnormally elevated REM sleep EMG (i.e., REM sleep without atonia) and dream mentation [5]. It became critical to accurately identify REM sleep without atonia once it became recognized that RBD is one of the earliest clinical manifestations of Parkinson’s disease and other related synucleinopathies (e.g., dementia with Lewy bodies and multiple systems atrophy) [6,7,8,9,10,11]. Previous longitudinal studies have reported that 50–70% of individuals with RBD eventually develop an overt synucleinopathy within 5–10 years of RBD diagnosis [12,13,14].

Efforts to quantify EMG in RBD have been carried out over the years. Lapierre and Montplaisir were first to quantify phasic mentalis EMG activity during REM sleep in RBD [15]. Bliwise et al. extended this initial report by Lapierre and Montplaisir describing a phasic electromyographic metric in patients with Parkinson’s disease [16]. Subsequently, more sophisticated computer algorithms have been developed across multiple groups. In a prior report by Fairley and Bliwise et al., 15 different EMG signal features were compiled after an exhaustive review of prior literature in the field [17]. These various automated quantitative approaches were compared relative to manual/visual review, and the accuracy of specific features tested. These features ranged from descriptive (e.g., skewness, kurtosis, and variance) to more complex algorithms involving nonlinear energy and spectral entropy. However, an intuitively appealing, fundamental approach was a simple ratio of high frequency to low frequency power density—an approach already used extensively in the analyses of electroencephalography (EEG) but not yet applied to EMG in this context [18]. Since EMG is composed of relatively high frequency signals, we elected to adopt this approach in the current work. Novel approaches to processing sleep signals generated from overnight sleep recordings have been introduced in recent years [18,19,20,21,22,23,24,25,26,27,28,29,30], many involving machine learning classification and/or power spectral analyses. Such newer techniques have led to important insights regarding clinical sleep disorders.

At the present time, clinical determination of REM sleep without atonia during overnight polysomnography still requires manual review and visual inspection by a registered polysomnographic scoring technician and/or board-certified sleep clinician. This manual approach is highly burdensome, time consuming, and prone to subjective errors. As such, scoring technicians do not routinely score each phasic EMG event, and ultimately, clinicians make a judgment call as to the binary presence or absence of REM sleep without atonia. Developing an automated method of identifying and quantifying REM sleep without atonia would accelerate the period of interpretation and reduce potential scoring bias. Additionally, quantification of REM sleep without atonia would provide a continuous, rather than binary, output variable by which to explore predictions of phenoconversion from RBD to parkinsonism. Thus, our goal was to develop and validate a more rigorous method to quantify REM sleep without atonia. Herein, we report an automated method of EMG quantification based upon ratios between high and low frequency (HF:LF) EMG spectral power and compare this to the gold standard visual scoring of REM sleep without atonia, manually labeled by a blinded scorer.

## 2. Materials and Methods

### 2.1. Participants and Polysomnography Recording

The overnight polysomnography data for this analysis was collected from an approved protocol performed according to the Declaration of Helsinki with approval of the VA Portland Health Care System Institutional Review Board (#3641). All participants provided verbal and written informed consent prior to participation.

**Figure 1:**
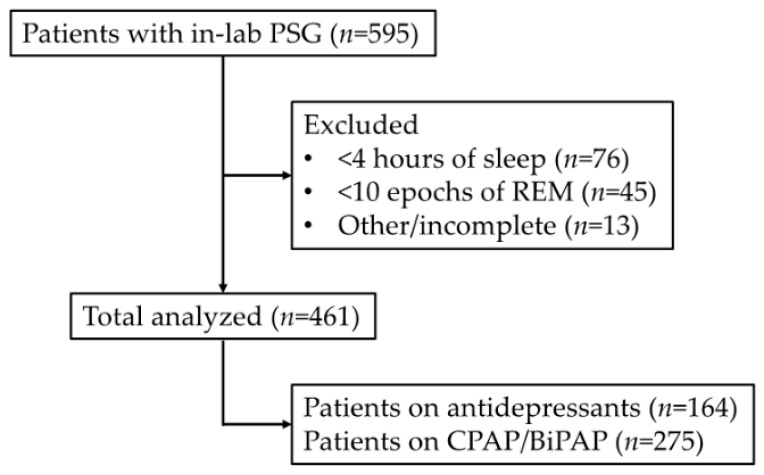
Schematic (CONSORT) overview of our patient population, exclusion criteria, and subsequent sub-analysis groups. Of the total *n* = 595 participants evaluated with in-lab polysomnography (PSG), we excluded *n* = 134 records (due to having <4 h of total sleep time, *n* = 76; <10 epochs of REM sleep, *n* = 45, or were otherwise incomplete, *n* = 13). Of these *n* = 461 participants, *n* = 164 and *n* = 275 were noted to be currently using antidepressant medications or were on CPAP/BiPAP during their PSG, respectively. Since antidepressant use and presence of untreated obstructive sleep apnea have both been associated with increased EMG tone during REM sleep, we wanted to examine these groups as separate subsets, given that these conditions might affect the accuracy of our algorithm.

Participants in this study were US Veterans and enrolled prospectively in a cross-sectional manner through the VA Portland Health Care System Sleep Clinic. A total of 595 Veterans enrolled with participants excluded based on (1) having <4 h of recorded sleep (*n* = 76), (2) having <10 epochs of recorded REM sleep (*n* = 45), and (3) were otherwise incomplete (*n* = 13). The remaining *n* = 461 participants were included in subsequent analyses (Figure 1). As previously described [31,32,33,34], reasons for referral to in-lab polysomnography included suspected obstructive sleep apnea, excessive daytime sleepiness, hypersomnia, insomnia, restless leg syndrome, and abnormal movements during sleep, with suspected obstructive sleep apnea and excessive daytime sleepiness being the most frequently cited.

All subjects completed in-laboratory, American Academy of Sleep Medicine-accredited technician-attended overnight video-polysomnography recordings using Polysmith (NihonKohden, Japan). Standard American Academy of Sleep Medicine parameters were collected, including electroencephalography (6 scalp electrodes), mentalis muscle EMG, bilateral tibialis anterior EMG, bilateral electrooculography, electrocardiography, peripheral blood-oxygen saturation, respiratory movement/effort (thorax and abdominal), airflow (nasal and oral), auditory (snoring), and body positioning (right side, left side, supine, prone) [1]. All EMG analyses described below were derived from the mentalis EMG channel. American Academy of Sleep Medicine-accredited polysomnographic technicians manually performed standard sleep staging for each 30 s epoch according to standard clinical criteria. Each 30 s epoch was scored as Wake, REM, or NREM stages N1, N2, and N3. All sleep staging was validated by a board-certified sleep physician.

All polysomnography records then underwent manual, visual-based scoring of phasic EMG events during REM sleep by a blinded, independent scorer. This training dataset was then used as the gold standard for direct comparison with our automated algorithm using HF:LF ratios.

### 2.2. HF:LF Analysis

Mentalis EMG, sampled at 200 Hz across the entire polysomnography recording, was analyzed using the spectral decomposition function in MATLAB’s signal processing toolbox. Estimates of power spectral density were computed via Welch’s method. Thus, we computed both absolute and relative powers of EMG in 2 s sliding windows which were overlapped by one-second, and which produced a time-varying power spectra with a 0.5 Hz resolution (1/2-s). Relative spectra were computed by dividing the absolute power spectra of each 2 s segment by the total spectral power of that segment.

From the second-by-second spectral density functions, we computed the ratio of the integral (sum) of EMG powers in the high and low frequency ranges using the formula:

(1) $$HF:LF\;\left(t\right)=\overset{55\;\mathrm{Hz}}{\underset{f=20}{\sum }}P_{EMG}(t,f)/\overset{20\;\mathrm{Hz}}{\underset{f=2}{\sum }}P_{EMG}\left(t,f\right)$$

where *P_EMG_* (*t*,*f*) is the spectral power density at time *t* and frequency *f* (*f* is between 0 and 55 Hz in 0.5 Hz steps).

Following the computation of HF:LF for every second of recorded EMG, we identified all the 30 s epochs of REM sleep and investigated the level of these indices with regards to the presence of phasic REM sleep without atonia. The following two methods were applied:

### 2.3. Epoch-Based HF:LF Analysis: Grouping Based on the Number of REM Sleep without Atonia Episodes per REM Epoch

In this method, the HF:LF for every REM epoch was summed and then considered individually. Every epoch of REM sleep was pooled together and separated according to the number of phasic REM sleep without atonia events that were identified during manual/visual review. Using this approach, we identified 3 groups: (E1) average HF:LF in all epochs with REM sleep without atonia; (E2) average HF:LF in every epoch of REM sleep with exactly 1 REM sleep without atonia event; and (E3) average HF:LF in every epoch of REM sleep with ≥2 REM sleep without atonia events.

### 2.4. Window-Based HF:LF Analysis: Compare All EMG-Index Values for Every Second

In this method, the HF:LF for every second of REM sleep was considered individually. Here we examined both every second of REM sleep without REM sleep without atonia and then specifically every REM sleep without atonia event using a 3 s window (average HF:LF in the 1 s before, during, and after). This produced two separate groups:

(W1), the HF:LF in every second of REM sleep with no REM sleep without atonia; and (W2), the average HF:LF in the 3 s window around every REM sleep without atonia event.

## 3. Results

### 3.1. Datasets

All 461 participants’ polysomnography analyzed met a priori inclusion criteria related to having a threshold level of total sleep time and REM sleep duration (i.e., >4 h and ≥10 REM epochs). The overall participant group (Table 1) was predominantly male, middle-aged, obese, and of white racial and non-Hispanic/Latino ethnicity. The majority of participants reported some college education (or greater) and were married living with their spouse/partner. Roughly 1/3 of the participant group reported exercising >90 min/week, had a self-reported history of traumatic brain injury or provisional post-traumatic stress disorder diagnosis based on the PCL-5 [35]. Finally, nearly half of the participant group endorsed the RBD1Q single-question related to dream enactment described by Postuma et al. [36] and a self-reported history of restless legs syndrome.

### 3.2. EMG Relative Spectral Power

**Figure 2:**
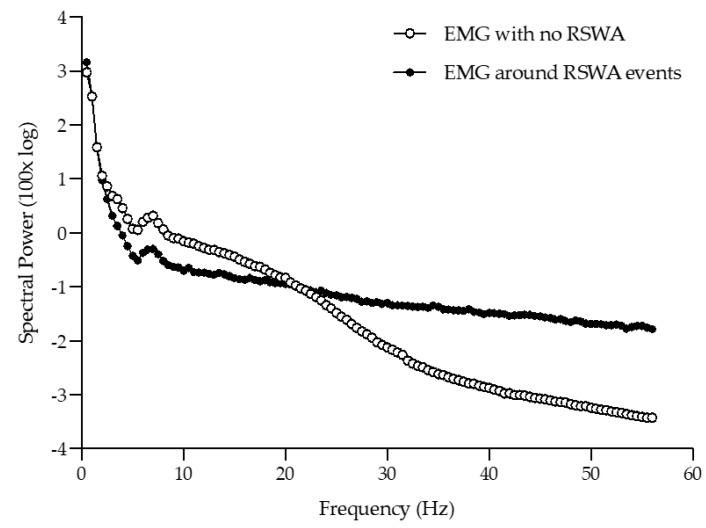
Overall median second-by-second EMG relative spectral power around periods of no REM sleep without atonia events and periods with an REM sleep without atonia event.

Relative spectral power within the EMG signal was computed on a second-by-second basis across all REM sleep epochs. The median value within all REM sleep epochs was then separated based on whether or not there was a recorded REM sleep without atonia event. These relative EMG spectral power data within periods of no REM sleep without atonia and periods of REM sleep without atonia (i.e., consisting of a 3 s window around the REM sleep without atonia event; 1 s before and 1 s after) are presented in Figure 2. The crossover point occurred between 20.5 and 21 Hz. This crossover provided the subsequent rationale to analyze the ratio between high and low frequency power (HF:LF).

### 3.3. Epoch-Based HF:LF Analysis

**Figure 3:**
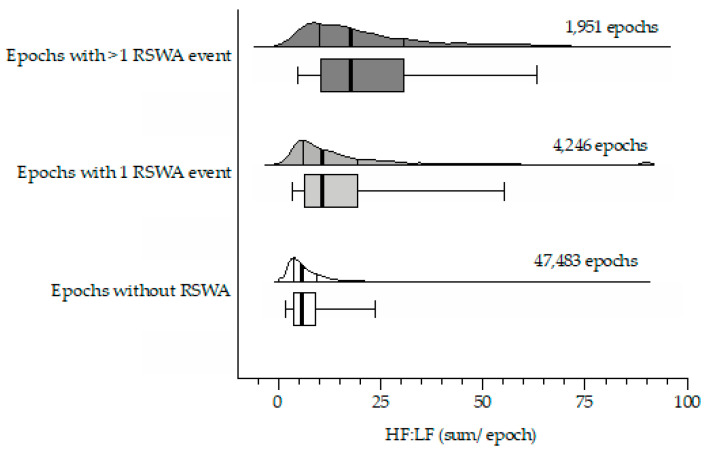
Epoch-based HF:LF analysis: horizontal violin plots (non-truncated/halved) with corresponding box-whisker plots illustrating the spread across Group E1 (open plot; average HF:LF in all epochs of REM sleep without REM sleep without atonia), Group E2 (light shaded plot; average HF:LF in every epoch of REM sleep with exactly 1 REM sleep without atonia event), and Group E3 (heavy shaded plot; average HF:LF in every epoch of REM sleep with ≥2 REM sleep without atonia events). The heavy solid line corresponds to the median value with the 25th and 75th percentile indicated by the bracketed lines/box outline. Whiskers indicate the 5th and 95th percentiles.

In this approach, the HF:LF per second was averaged across every epoch of REM sleep and considered individually (Figure 3). This produced a total of 53,680 epochs of REM sleep, of which there were 47,483 epochs with no REM sleep without atonia events (Group E1), 4246 epochs with exactly 1 REM sleep without atonia event (Group E2), and 1951 epochs with ≥2 REM sleep without atonia events (Group E3). Average HF:LF for groups E1, E2, and E3 were 8.38 ± 9.42, 16.68 ± 17.36, and 23.52 ± 18.35, respectively. Median (25th–75th percentile) HF:LF for groups E1, E2, and E3 were 5.69 (3.66–9.40), 10.80 (6.30–19.47), 17.73 (10.04–30.84), respectively. Statistical power considering each REM epoch individually is likely exaggerated (omnibus ANOVA: *p* < 0.0001, F (2, 53,677)), with Tukey’s posthoc analysis illustrating significant differences between each of the comparisons (all *p* < 0.0001).

The same comparison was made within two separate sub-analyses, considering participants who were on/off CPAP/BiPAP during their polysomnography, and considering participants who were/were not currently taking antidepressants at the time of their polysomnography. There was *n* = 275 (corresponding to 28,229 REM sleep epochs) and *n* = 186 (corresponding to 21,106 REM sleep epochs) participants on and off CPAP/BiPAP, respectively. Similarly, there was *n* = 164 (corresponding to 18,705 REM sleep epochs) and *n* = 286 (corresponding to 33,794 REM sleep epochs) participants on and off antidepressant medications, respectively. The subanalyses did not produce different distributions or relative numbers of epochs per grouping (i.e., groups E1, E2, E3) compared to the whole group analysis (data not shown).

### 3.4. Window-Based HF:LF Analysis

**Figure 4:**
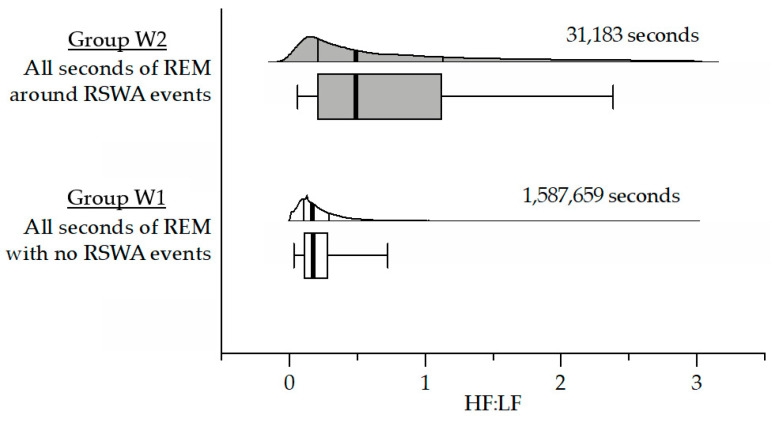
Window-based HF:LF analysis: horizontal violin plots (non-truncated/halved) with corresponding box-whisker plots illustrating the HF:L:F spread across Group W1 (open plot; the HF:LF in every second of REM sleep with no REM sleep without atonia), and Group W2 (light shaded plot; the average HF:LF in the 3 s window around every REM sleep without atonia event). The heavy solid line corresponds to the median value with the 25th and 75th percentile indicated by the bracketed lines/box outline. Whiskers indicate the 5th and 95th percentiles.

In this approach, the HF:LF for every second of REM sleep was computed and considered individually using a 1 s window (Figure 4). This produced a total of 1,618,842 s of REM sleep, of which there were 1,587,659 s with no REM sleep without atonia events (Group W1), and 31,183 s of REM sleep around REM sleep without atonia events (Group W2). Average HF:LF for Groups W1 and W2 were 0.26 ± 0.30, and 0.77 ± 0.72, respectively. Median (5th-75th percentile) HF:LF for Group W1 and Group W2 were 0.17 (0.10–0.29), and 0.49 (0.21–1.13), respectively. Statistical power considering each second of REM sleep individually is likely exaggerated yet significant via a two-tailed unpaired *t*-test (*p* < 0.0001; *t* = 125.3, df = 31,390) and F-test comparing variances (*p* < 0.0001; F = 5.89, DFn = 31,182, Dfd = 1,587,658).

As in the first method, epoch-based HF:LF analysis, the same comparison was made considering participants who were on/off CPAP/BiPAP during their polysomnography, and considering participants who were/were not currently taking antidepressants at the time of their polysomnography. There was *n* = 275 (corresponding to 848,141 s of REM sleep) and *n* = 186 (corresponding to 639,543 s of REM sleep) participants on and off CPAP/BiPAP, respectively. Similarly, there was *n* = 164 (corresponding to 560,532 s of REM sleep) and *n* = 286 (corresponding to 1,022,332 s of REM sleep) participants on and off antidepressant medications, respectively. The subanalyses did not produce different distributions or relative numbers of seconds per grouping (i.e., Groups W1 or W2) compared to the whole group analysis (data not shown).

## 4. Discussion

We sought to validate a novel automated algorithm to identify and quantify phasic REM sleep without atonia events in human polysomnography records. Power spectral analyses of manually/visually scored events showed exceptionally strong correspondence, suggesting that the HF:LF approach developed here represented an extremely robust algorithm. We tested this with two separate analyses: (1) epoch-based HF:LF analysis that calculated the HF:LF on a conventional epoch-by-epoch basis (e.g., 30 s epochs are standard for sleep staging in human polysomnography recordings), and (2) window-based HF:LF analysis that calculated HF:LF on a second-by-second basis. Both analyses revealed high comparability to the manual, visually based gold standard scoring rubric, and analyses indicated that our method was highly accurate in discriminating phasic REM sleep without atonia from baseline REM sleep EMG tone.

These two methods that we developed and validated were both highly accurate, and importantly, are complementary to each other. Our rationale to presenting both approaches derives from the fact that, in the study of human sleep, there has been little consensus on the optimal time base with which to analyze the EMG signal. Clinically, scoring set forth by the American Academy of Sleep Medicine guidelines determine human sleep staging at no shorter than 30 s at a time (i.e., 30 s epochs). The epoch-based HF:LF analysis remains faithful to this and although using a sophisticated parametric approach to signal processing (i.e., the HF:LF ratio computed on an individual second-to-second basis), this approach still resolves into being non-parametric, classifying 30 s REM sleep epochs into those with 0 events, 1 event, or 2 or more events. This non-parametric paradigm was retained to maintain translational compatibility with how human sleep recordings are clinically analyzed visually.

In contrast to the epoch-based HF:LF analysis, the window-based HF:LF analysis establishes the relevant time base for application of the HF:LF ratios on a 1 s basis. This approach allows for a more fully parametric use of the measured ratios, because it makes no assumptions about the number of single seconds with activity that are meaningful. Furthermore, the increased temporal resolution of the window-based HF:LF analysis might be useful during sleep stage transitions between epochs and/or during sleep studies using animal models, which are not constrained by the 30 s epoch rule. In fact, the newly published statement from the International REM Behavior Disorder working group [37] has suggested that just such micro-epoch scoring is likely to have the highest yield clinically and produce the more consistent results across laboratories. Although full analyses of clinical material are beyond the scope of the current paper, a potential and immediate benefit of the higher temporal resolution and more parametric approach is seen in Figure 3. These data highlight the benefit of more fine-grained resolution in the analysis of the EMG signals, in this case for determination of cut-point frequency defining the presence or absence of activity.

The epoch- and window-based HF:LF analyses each reveal different aspects of the measurement of the EMG in human sleep. The epoch-based HF:LF analysis allows immediate translation of this particular signal processing strategy to an enormous body of literature that has approached the analyses of EMG signals in sleep using visual techniques. The window-based HF:LF analysis will potentially pave the way for future work in this area. As such, the two analytic approaches are complementary to each other and reveal a more complete story than either could tell alone.

Although other approaches to digitization of surface EMG signals recorded during sleep have been developed, validation of such approaches typically have relied upon evaluating their utility in the service of diagnostic relevance [21,38,39,40,41,42,43]. Ultimately, such approaches confound complex issues of medical diagnoses, such as incidence, positive predictive value, and sensitivity/specificity of a disease, with the particular signal processing approach being tested. By contrast, our analyses may be considered more elemental and have focused solely on validating the HF:LF approach with the judgments of expert visual scorers about those signals. Such an approach makes fewer epidemiologic assumptions about how well a particular digital feature operates, and maintains a strict focus only on technologic, rather than clinical, utility. Nonetheless, we evaluated the impact of several additional factors impacting EMG activity in sleep.

In clinical practice, EMG tone during REM sleep can be affected by several factors. One of the most common conditions that can cause increased EMG tone during REM sleep is the use of anti-depressants, especially within the selective serotonin reuptake inhibitor (SSRI) class [44,45]. To this end, we assessed whether our HF:LF algorithm could distinguish REM sleep without atonia within those subjects on anti-depressants, versus those not on anti-depressants, and found that the algorithm was still highly accurate and comparable to manual visual scoring. Another common condition that can cause increased EMG tone during REM sleep is obstructive sleep apnea, in which increased apneas during REM sleep can cause muscle artifact through snoring and other respiratory-related movements of the head. In order to address sleep apnea as a potential confound, we assessed whether our HF:LF algorithm could distinguish REM sleep without atonia within those subjects with mild, moderate, and severe sleep apnea, versus those without sleep apnea, and found that the algorithm was still highly accurate in identifying REM sleep without atonia regardless of sleep apnea status.

Strengths of this study include a large sample size comprised of over 450 polysomnographic records yielding over 50,000 epochs of REM sleep for analysis. Additionally, our dataset with manual visual scoring of REM sleep without atonia phasic events in these polysomnography records, in addition to American Academy of Sleep Medicine-standard scoring, represents a valuable gold standard with which to compare other automated methods. Finally, the ratio approach is novel, with strong rationale based upon naturally occurring cutoffs in the power spectra from gold standard REM sleep without atonia versus non-REM sleep without atonia events and may serve to accentuate existing differences between REM sleep without atonia and non REM sleep without atonia EMG signal.

Limitations of our analysis include the following considerations. While the dataset is large, it was obtained from a single site. As this site is an American Academy of Sleep Medicine-accredited sleep laboratory and studies were obtained for clinical indications and not research, no additional EMG leads were acquired beyond the standard chin and leg leads. The addition of arm leads and other muscles may increase the sensitivity for detecting REM sleep without atonia and diagnosis of RBD [46]. Finally, our HF:LF algorithm was validated against gold standard scoring of phasic, and not tonic, events only. As the REM sleep without atonia events in this dataset contained nearly four times the number of phasic events compared to tonic events, this may be a minor limitation. However, some have postulated that sustained tonic EMG activity essentially represents a cumulative summation of shorter duration phasic events [47], and basic science studies have suggested that phasic activity may represent the most fundamental neurobiological substrate of the neurochemical control of REM [48,49,50]. Nonetheless, there remains a caveat about generalization of our algorithm to tonic events and other cohorts/datasets that may have more tonic activity. Lastly, we note that the proposed signal analytic approaches (i.e., HF:LF ratios) are not inherently novel from a pure signal processing perspective. As described, this mathematical approach has a considerable history of usage. However, we note that the novelty of the proposed analysis lies in the application and potential clinical utility. This approach, while simple, mirrors the human visual experience and sets the stage for future advanced proposals that leverage machine learning and other methodologies beyond the scope of the current report. Additional analytical progressions that combine these signal processing approaches, and clinical application, will include how to define performance of the models. This may be based comparisons to human scoring on a second-by-second basis, agreement on a 30 s epoch basis, agreement on binary diagnosis, or even examining the extent/quantification of RSWA as a continuous variable.

In summary, these data indicate that our automated HF:LF ratio approach to EMG quantification during human sleep recording is practical, feasible, and may provide a much-needed clinical tool for screening of phasic REM sleep without atonia events as relevant to diagnosis of REM sleep behavior disorder and eventual phenoconversion to parkinsonism [51].

## 5. Conclusions

We report a highly accurate approach to detect and quantify surface EMG activity during sleep using a large dataset of overnight polysomnography containing over 50,000 epochs of REM sleep from over 450 individuals. This digitized, signal processing method utilizes the ratio of high frequency to low frequency (HF:LF) spectral power and validated this method against expert human scorer interpretation of EMG signal. Data demonstrate a significant association between visual interpretation and the spectrally processed signals, indicating a highly accurate approach to detecting and quantifying abnormally high levels of EMG activity during REM sleep. These data indicate that our automated approach to EMG quantification during human sleep recording is practical, feasible, and may provide a much-needed clinical tool for screening of disorders with elevated EMG tone during REM sleep, such as REM sleep behavior disorder and parkinsonism.

## Figures and Tables

**Table 1:** Demographic and anthropometric variables.

	Whole Group
	*n* = 461
Age, years	53.9 ± 15.9
Sex, male	92.0%
Height, cm	177.2 ± 7.8
Weight, kg	102.3 ± 21.1
BMI, kg/m^2^	32.6 ± 6.6
Race, white	83.9%
Ethnicity, not Hispanic or Latino	90.2%
Education, at least some college	79.0%
Marital status, married/partnered	62.5%
Living situation, spouse/partner	63.8%
Exercise, >90 min/week	28.0%
TBI, yes	20.8%
PTSD, yes	30.6%
RBD1Q dream enactment, yes	43.4%
RLS, yes	44.3%
Snore, yes	88.3%
CPAP/BiPAP, yes	59.7%
Antidepressant use, yes	35.6%

Data are presented as mean ± standard deviation or as a % of the total sample size. BMI, body mass index; TBI, traumatic brain injury; PTSD, post-traumatic stress disorder; RBD1Q, previously published single question related to dream enactment; RSL, restless legs syndrome; CPAP/BiPAP, continuous positive airway pressure/bidirectional positive airway pressure.

## Data Availability

A de-identified, anonymized dataset will be created and shared, and released once institutional approvals are in place. Where practicable, sharing will take place under a written agreement prohibiting the recipient from identifying or re-identifying (or taking steps to identify or re-identify) any individual whose data are included in the dataset. However, it is permissible for final datasets in machine-readable format to be submitted to and accessed from PubMed Central (and similar sites) provided that care is taken to ensure that the individuals cannot be re-identified using other publicly available information. De-identified data sets will be maintained locally on VA computer drives that are regularly backed up and password protected until central data repositories become available for long-term storage and access. Until that time, the data sets will be available by request to any interested investigators together with a data dictionary to enable interpretation of the data set.
